# Life-style and Other Characteristics of Radiation Workers at Nuclear Facilities in Japan: Base-line Data of a Questionnaire Survey

**DOI:** 10.2188/jea.12.310

**Published:** 2007-11-30

**Authors:** Motoi Murata, Toshio Miyake, Yasushi Inoue, Sumio Ohshima, Shin-ichi Kudo, Takesumi Yoshimura, Suminori Akiba, Toshiro Tango, Yasuhiko Yoshimoto, Yukiko Shimizu, Tomotaka Sobue, Shizuyo Kusumi, Tamiko Iwasaki, Chikao Yamagishi, Hiromichi Matsudaira

**Affiliations:** 1 Radiation Effects Association.; 2 University of Occupational and Environmental Health.; 3 Kagoshima University.; 4 National Institute of Public Health.; 5 National Institute of Radiological Sciences.; 6 Radiation Effects Research Foundation.; 7 National Cancer Center Research Institute.

**Keywords:** radiation worker, low dose radiation, cancer mortality, confounding factor, life-style

## Abstract

To examine confounding on the risk assessment of the radiation workers at nuclear facilities in Japan, a questionnaire survey of their characteristics such as life-style and occupational history was performed for 54,369 male and 470 female workers who were currently engaged in the job and valid answers were obtained from 48,281 males and 428 females. In order to know whether these characteristics were different among different dose groups, the Mantel extension statistical test was performed only for male respondents, with cumulative radiation doses stratified into 5 classes. Increasing trend according to the increasing doses was statistically significant for the percentages of tobacco smokers and of heavy smokers. It was also the case for heavy alcohol drinkers. Percentages of workers who were engaged in jobs dealing with specific toxic materials were also increasing in the higher dose groups. On the other hand, percentage of workers who underwent the X-ray examination of the upper digestive tracts or other radiological examinations tended to be lower in higher dose groups. These results indicate that characteristics of radiation workers such as life-style are different among dose groups and thus may play a role as a confounding factor in the statistical relation between the radiation doses and cancer mortalities.

A cohort study of radiation workers at nuclear power stations and other nuclear facilities in Japan is underway to obtain scientific data on health effects (mainly on malignant neoplasms) of low dose and low dose rate radiation in humans.^1^ However, factors other than exposure to radiation including life-style habits, such as smoking and drinking, and exposure to certain toxic substances are also known to be responsible in part for malignant neoplasms.^2^ In addition, radiological examinations are widely conducted for medical purposes in Japan.^3^ If these factors are correlated to the levels of radiation exposure among radiation workers, they may be possible confounding factors in the ongoing radiation epidemiologic study.

Some reports on similar studies in radiation epidemiology in other countries have discussed the possible effects of such confounding factors as smoking in the analysis of their results.^4^^,^^5^ The International Agency for Research on Cancer (IARC), which is promoting international collaborative studies to assemble results of similar studies in each participating country, has recommended a study protocol in which the effects of confounding factors such as the socio-economic status are adjusted for.^6^

When the present radiation epidemiologic study was begun in 1990, analysis of confounding factors, such as the life-style habits of the subjects, was not included in the initial study design.^1^ It was felt, however, imperative to examine the effects of confounding factors when the study results were analyzed and discussed. Then, a questionnaire study on certain life-style habits and other factors was conducted on currently actively employed radiation workers.

## MATERIALS AND METHODS

Plans to conduct a questionnaire survey were approved by the Director-General of the Management and Coordination Agency (presently, Ministry of Public Management, Home Affairs, Posts and Telecommunications) in accordance with the Statistics Report Adjustment Law (No.148, 1952). The Ministry of Science and Technology Agency (STA, presently, the Ministry of Education, Culture, Sports, Science and Technology, or MEXT) undertook the organization of the study, and entrusted the development of the design and conduct of the study as well as the analysis of the collected data to the Radiation Effects Association (REA). Institute of Radiation Epidemiology (IRE), the research arm of REA, took the responsibility for these tasks, with the support of a study team of epidemiologists.

### Study Population

In Japan, the Radiation Dose Registration Center (RADREC) was established in REA in 1978 to register radiation doses of almost all radiation workers at nuclear power plants and associated facilities for the purpose of individual dose management. All the dosimetry records of those workers from back to 1957 were reported from the respective nuclear facilities and maintained in a computer file. Presently dose data of about 60,000 workers are annually registered, excepting those (presumably about 360) who are working at nuclear power plants installed for research purpose in some universities. Details of the registration system are described in our previous paper.^1^ Our separately conducted cohort study (T. Iwasaki et al, under submission) was based on 176,000 workers who were either currently engaged in or already retired from the radiation works. The present study covered only those currently engaged in the radiation work.

### Questionnaire

The questionnaire was a self-administered type, and filled out personally by the workers including his/her name, date of birth, central registration number at RADREC and date of entry.

Questions on life-style habits included tobacco smoking and alcohol intake, which have been demonstrated to be related to certain cancers in a number of epidemiologic studies.^7^^,^^8^ Also included was the consumption of non-alcoholic beverages such as Japanese green tea, which has recently been strongly suggested as having certain preventive effects against cancers.^9^ Dietary habits, although deemed important,^10^ were excluded because this questionnaire was intended not to be too complicated.

Since certain kinds of chemicals are considered to be carcinogenic,^11^^,^^12^ past history of employment in specific hazardous works dealing with such materials was examined. Exposure to medical radiation was also studied, because the dose from this source could not be ignored in the examination of the relationship between cancer and occupational radiation exposure.

Thus questionnaires adopted were as follows.

1) Tobacco consumption - Does the respondent smoke, and if so, since when? How many cigarettes are smoked per day? How long ago was smoking discontinued?
2) Alcohol consumption - Does the respondent drink alcohol, and if so, since when? What kinds of alcoholic beverages are consumed, and what is the daily consumption? How long ago was drinking discontinued?
3) Consumption of tea, coffee, etc. - Frequency of drinking Japanese green tea, black tea, oolong tea, and coffee.
4) History of work involving hazardous materials - History of special health examination programs for those with an occupational history of handling asbestos, dust, chrome, nickel, arsenic, organic solvents, benzene, aromatic amines, coke, asphalt, and other sources of microscopic particulates, etc.
5) History of medical exposure to radiation - History of undergoing X-ray mass examination of the upper digestive tracts and other medical examinations using X-rays or other nuclear medicine procedures during the year prior to entry into the survey.

### Implementation of the study

Members of several organizations, who are specialized in field studies, engaged in the distribution and collection of the questionnaires. Delivery of questionnaires to the study subjects was made via the management of nuclear facilities during the period 1997 through 1999.

IRE staff members briefed all of these organizations in order to ensure uniformity of conduct of the survey at each of the total of 32 nuclear facilities. The operation manual for the survey was explained to relevant members of these organizations for full understanding of the study procedures. In addition, an explanatory meeting was held for those to be involved directly with the execution of the survey at the respective facilities so that they also thoroughly understand the study procedures, thereby preventing methodological differences among different facilities.

The questionnaire was distributed with a letter from STA requesting the cooperation and understanding of the study subjects along with a leaflet, which described the objectives and contents of the study. The leaflet also explained that participation in the study was entirely voluntary and solicited the maximum possible cooperation from the study subjects. In addition, a toll free telephone service was arranged so that study subjects could directly contact IRE for any questions they may have.

After each study subject had filled out the questionnaire and sealed it in a reply envelope, the questionnaire was collected by sending it back either via the original distribution channel or directly to IRE by mail.

### Database

The returned questionnaires were checked manually, and those deemed error-free were coded and entered into the computer file. Errors included no answers (blank), illegible writing, impossible or inconsistent answers, etc. No inquiries were made to the respondents to clarify answers. Instead, rules for handling inappropriate answers to each of the questions were established by the study team, and answers were processed accordingly and entered into the computer file.

### Dose data

Individual annual doses (effective dose equivalent) provided by RADREC to IRE were used to compute the cumulative radiation dose for each subject from the year of commencement of radiation work to the end of March 1999. The classification of the cumulative dose groups was the same as that adopted for the analysis of the health follow-up study,^1^ namely stratification into five groups; 0-9, 10-19, 20-49, 50-99, and 100+ mSv. The subjects without the dose records were excluded from the statistical analysis described below.

### Statistical analysis

The answers were cross-tabulated by age-class and cumulative dose group. The relationship between cumulative dose and positive rate of a certain factor (e.g. the proportion of smokers) was examined in the following way.

The five dose groups were scored from 1 to 5, respectively, from the lowest to the highest doses. The ages of the respondents, as of April 1, 1998, were divided into five groups; 20-29, 30-39, 40-49, 50-59, and 60+ years and also scored from 1 to 5, respectively.

For each question, respondents were classified dichotomously by their answer, e.g. into smokers and others. Those who did not answer correctly were excluded from the total number. For those questions which required answer to be given in numbers, e.g. the number of cigarettes smoked per day, the answers were also divided into two groups at an appropriate cutting point.

In order to assess the amount of alcohol drunk by individual subjects, the average ethanol intake per day was calculated for current and former drinkers by the following conversion formula and summing all kinds of alcoholic beverages.

(No. of drinks annually) x (intake volume/time) x (ethanol content in unit of each alcoholic beverage) / 365, where the ethanol content is defined as 23gr per go (0.18 lit.) of Japanese sake, and one go of sake equals a large bottle of beer, two singles of whisky, two glasses of wine, or two cups of shochu or inexpensive Japanese distilled spirits in terms of ethanol content.

### (1) Examination of interaction with age at the time of response

When the relationship of the positive rate of a certain factor (question) to the cumulative doses or its regression against dose group scores apparently differed among age-classes, an interactive effect of the dose and age class was suspected for the positive rate. Then the following logistic regression model was employed to determine whether there was any interaction between the two.

The CATMOD procedure for statistical analysis developed by SAS (SAS Institute, Japan)^13^ was used to estimate the coefficient *β*_12_ of the interaction term of the equation

$$\ln(p/(1-p))=\beta_{0}+\beta_{1}\;X_{1}+\beta_{2}\;X_{2}+\beta_{12}\;X_{1}\;X_{2},$$

where p represents the positive rate of a factor (e.g. smoking rate), X_1_ represents scores for the cumulative dose classes, X_2_ represents scores for the age classes, X_1_ X_2_ represents interaction of both scores, and *β*s are regression coefficients.

In cases where the coefficient *β*_12_ proved to be statistically significant (p<0.05), it was judged that the interaction between cumulative dose and age at the time of response was significant, or that the relationship between the positive rate of a certain factor (e.g. the smoking rate) and cumulative dose differed among age-classes.

### (2) Relationships with cumulative doses

The Mantel extension statistical test for trend analysis^14^ was performed using the FREQ procedure on the SAS software in order to examine whether the positive rate of a certain factor was associated with cumulative dose. The test was performed for each age class separately if a significant interaction was recognized between dose and age at the time of response with the logistic regression analysis, while it was applied after adjusting for the scores of age classes if the interaction was insignificant. When the trend of the positive rate of a certain factor with increasing doses was statistically significant in the two-sided test, it was judged that higher cumulative dose groups had higher (or lower) positive rates.

## RESULTS

### (1) Reply rate

The total numbers of questionnaires successfully distributed and collected were 55,271 and 50,526, respectively (males and females inclusive). Exclusion of questionnaires with various kinds of invalid reply resulted in a total number of 49,065 valid replies. Furthermore some of the respondents were found to have not actually engaged in radiation works. The total number of study subjects who had cumulative dose records was 54,839 (54,369 males and 470 females) and that of respondents who gave valid replies was 48,709 (48,281 males and 428 females). Since the number of female respondents was too small, only male respondents were enrolled for the statistical analysis in the following section. The reply rate by cumulative dose group and age-class is shown only for males in Tables 1 and 2, respectively. The reply rate was 88.8% on the average and was slightly but significantly higher in the higher cumulative dose groups (*χ*^2^ =236.2, d.f.=4, p<0.0001). When examined by age-classes, those of the age-class of 20-29 years yielded a relatively poor reply rate.

**Table 1.** Number of male study subjects and participants in questionnaire survey by dose class.

Dose categories (mSv)	Study subjects	Participants	%
0-9	35,532	31,040	87.4
10-19	5,582	5,060	90.6
20-49	6,786	6,182	91.1
50-99	3,780	3,475	91.9
100+	2,689	2,524	93.9
Total	54,369	48,281	88.8

*χ*^2^ test for contingency: *χ*^2^ =236.2, d.f.=4, p<0.0001

**Table 2.** Number of male study subjects and participants in questionnaire survey by age class at the time of response.

Age classes (yrs)	Study subjects	Participants	%
20-29	15,866	13,846	87.3
30-39	14,786	13,160	89.0
40-49	13,488	12,096	89.7
50-59	8,312	7,451	89.6
60+	1,917	1,728	90.1
Total	54,369	48,281	88.8

*χ*^2^ test for contingency: *χ*^2^ =57.9, d.f.=4, p<0.0001

### (2) Results of the statistical analyses

#### Age distribution at the time of response

Table 3 shows the cross-tabulation of male respondents by dose group and age class. The mean age at the time of response was 38.1 years. It is apparent that the higher the dose, the older the age of workers (*χ*^2^ =4097.7, d.f.=16, p<0.0001). Accordingly, in the following statistical analyses, it was necessary to either adjust for age or to separate the age classes.

**Table 3.** Number of male participants in questionnaire survey by age class and dose category.

		Dose categories (mSv)					
Age classes (yrs)	0-9	10-19	20-49	50-99	100+	Total	(%)
20-29	11,416	1,249	959	202	20	13,846	(28.7)
30-39	8,004	1,596	2,013	1,027	520	13,160	(27.3)
40-49	6,355	1,340	1,946	1,334	1,121	12,096	(25.1)
50-59	4,192	746	1,056	747	708	7,451	(15.4)
60+	1,073	129	206	165	155	1,728	(3.4)
Total	31,040	5,060	6,182	3,475	2,524	48,281	(100.0)
Mean age (yrs)	36.3	38.3	40.6	43.6	46.6	38.1	

*χ*^2^ test for contingency: *χ*^2^ =4097.7, d.f.=16, p<0.0001

### Tobacco smoking

**Figure 1.**
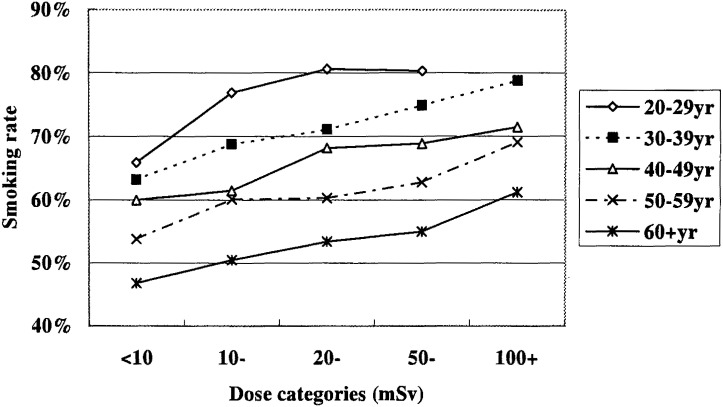
Percentages of current tobacco smokers in male workers by age class and dose category. Interaction of age and dose classes on the percentage; p<0.001. Positive trend with increasing doses; p<0.001 in all the age classes.

The number of subjects by current status of tobacco smoking is shown for different dose groups in Table 4. It can be seen that higher dose groups had higher percentage of current smokers. Furthermore, as is apparent in Figure 1, those in younger age-classes showed higher rates of current smoking. The total percentage of current smokers was 68.1, 66.6, 63.5, 57.7 and 49.8% in age classes 20-29, 30-39, 40-49, 50-59 and 60+ years, respectively.

**Table 4.** History of tobacco smoking in male workers of different dose categories.

Smoking history		Dose categories (mSv)				
	0-9	10-19	20-49	50-99	100+	Total
Current smoker	18,382	3,237	4,080	2,277	1,687	29,663
	(59.2)	(64.0)	(66.0)	(65.5)	(66.8)	(61.4)
Past smoker	4,005	714	862	535	389	6,505
	(12.9)	(14.1)	(13.9)	(15.4)	(15.4)	(13.5)
Never-smoker	7,356	863	945	463	274	9,901
	(23.7)	(17.1)	(15.3)	(13.3)	(10.9)	(20.5)
Unknown	1,297	246	295	200	174	2,212
	(4.2)	(4.9)	(4.8)	(5.8)	(6.9)	(4.6)
Total	31,040	5,060	6,182	3,475	2,524	48,281
	(100.)	(100.)	(100.)	(100.)	(100.)	(100.)

percentages in parentheses.

As is also clearly seen in this Figure, the current smoking rate was increased in the higher dose groups. This trend was tested for individual age classes separately, because a significant interaction was recognized, with the logistic regression analysis, between age class and dose group with respect to the smoking rate (p<0.001). As a result of the Mantel extension test, the current smoking rate was found to be significantly higher with increasing dose (p<0.001 in all age classes).

With respect to ages at commencement of smoking in the current smokers, when examined by the age classes at the time of response, those in higher dose groups tended to have begun smoking at younger age (data not shown). Since interaction between the dose and age classes was not significant with the logistic regression analysis (p=0.347), the trend of the percentage of those who started smoking before age 20 years to be higher in relation to increasing doses was tested after adjustment for the age classes at the time of response, and was demonstrated to be statistically significant (p<0.001).

Table 5 shows, current and past smokers inclusive, that those in higher dose groups smoked a greater number of cigarettes per day. The trend for the percentage of those who smoked 25 or more cigarettes per day to be higher in relation to increasing doses was tested in individual age classes separately, since a significant interaction with respect to the daily tobacco consumptions was recognized between the dose groups and the age-classes at the time of response. The trend was statistically significant in all age classes (data not shown). The similar tendency was found for the smoking index, which was calculated by multiplying number of packs (20 pieces) of cigarettes smoked per day and number of smoking years for the current and former smokers (data not shown).

**Table 5.** Daily tobacco consumption in current and past smokers of different dose categories.

Number of cigarettes /day		Dose categories (mSv)				
	0-9	10-19	20-49	50-99	100+	Total
<10	680	77	82	36	31	906
	(3.0)	(1.9)	(1.7)	(1.3)	(1.5)	(2.5)
10-19	4,935	780	848	454	291	7,308
	(22.0)	(19.7)	(17.2)	(16.1)	(14.0)	(20.2)
20-29	10,883	1,930	2,486	1,329	952	17,580
	(48.6)	(48.8)	(50.3)	(47.3)	(45.9)	(48.6)
30-39	3,781	753	989	632	463	6,618
	(16.9)	(19.1)	(20.0)	(22.5)	(22.3)	(18.3)
40+	1,933	383	488	327	312	3,443
	(8.6)	(9.7)	(9.9)	(11.6)	(15.0)	(9.5)
Unknown	175	28	49	34	27	313
	(0.8)	(0.7)	(1.0)	(1.2)	(1.3)	(0.9)
Total	22,387	3,951	4,942	2,812	2,076	36,168
	(100.)	(100.)	(100.)	(100.)	(100.)	(100.)

percentages in parentheses.

### Alcohol drinking

The percentage of current alcohol drinkers was 80.1% in the total population, and 75.3, 82.2, 82.8, 82.2 and 74.3% in age classes at the time of response 20-29, 30-39, 40-49, 50-59 and 60+ years, respectively. In contrast to the smoking rate, the drinking rate showed no clear age dependency. Furthermore, as shown in Table 6, the rate of alcohol drinking did almost not differ among dose groups. This was confirmed by the trend analysis: p=0.618 for the interaction between age and dose classes with the logistic regression analysis, and p=0.377 for the trend with increasing dose after adjustment for age class in the Mantel extension test. Regarding the varieties of alcoholic beverages taken, about 87% of the respondents answered “beer”, followed by 48% of “Japanese sake”, about 45% of “shochu”, 42% of whisky and other spirits, and 23% of wine.

**Table 6.** Alcohol drinking habit in male workers of different dose categories.

Drinking habit		Dose categories (mSv)				
	0-9	10-19	20-49	50-99	100+	Total
Current drinker	23,640	3,934	4,726	2,661	1,895	36,856
	(76.2)	(77.7)	(76.4)	(76.6)	(75.1)	(76.3)
Past drinker	606	113	158	94	77	1,048
	(2.0)	(2.2)	(2.6)	(2.7)	(3.1)	(2.2)
Never-drinker	5,509	769	980	525	354	8,137
	(17.7)	(15.2)	(15.9)	(15.1)	(14.0)	(16.9)
Unknown	1,285	244	318	195	198	2,240
	(4.1)	(4.8)	(5.1)	(5.6)	(7.8)	(4.6)
Total	31,040	5,060	6,182	3,475	2,524	48,281
	(100.)	(100.)	(100.)	(100.)	(100.)	(100.)

percentages in parentheses.

**Figure 2.**
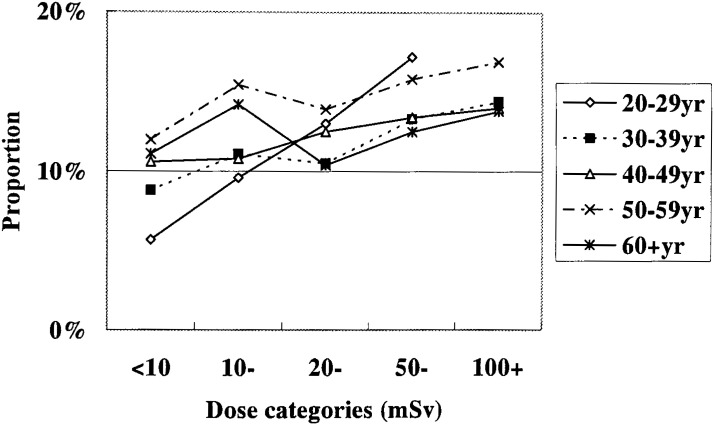
Percentages of male workers who drink 69 or more grams of ethanol per day by age class and dose category. Interaction of age and dose classes on the percentage; p<0.001. Trend with the doses; p<0.001 in the age classes of <30, 30-39 and 40-49; p=0.003 in the age class of 50-59; p=0.501 in the age class of 60+.

Table 7 shows distribution of average amount of ethanol intake per day for the current and former drinkers inclusive in different dose groups. It shows that those in higher dose groups tended to be heavier drinkers. The statistical test of whether respondents in higher dose groups tended to be heavier alcohol drinkers was conducted for the percentage of those drinking 69 gr or more/day. It was done for different age-classes separately, because the interaction between the dose and age classes was significant (p<0.001). The trend was found to be significant in all but the highest age classes (Figure 2).

**Table 7.** Amount of alcohol intake per day in current and past drinkers of different dose categories.

Ethanol intake (grams / day)		Dose categories (mSv)				
	0-9	10-19	20-49	50-99	100+	Total
<23	13,420	1,998	2,293	1,207	821	19,739
	(55.3)	(49.4)	(46.9)	(43.8)	(41.6)	(52.1)
23-45	4,089	765	941	495	412	6,702
	(16.9)	(18.9)	(19.3)	(18.0)	(20.9)	(17.7)
46-68	2,184	414	522	321	214	3,655
	(9.0)	(10.2)	(10.7)	(11.7)	(10.9)	(9.6)
69+	2,530	546	709	466	343	4,594
	(10.4)	(13.5)	(14.5)	(16.9)	(17.4)	(12.1)
Unknown	2,023	324	419	266	182	3,214
	(8.3)	(8.0)	(8.6)	(9.7)	(9.2)	(8.5)
Total	24,246	4,047	4,884	2,755	1,972	37,904
	(100.)	(100.)	(100.)	(100.)	(100.)	(100.)

percentages in parentheses.

The respondents in higher dose groups also tended to have started drinking at significantly younger age when examined after adjustment for age class (p<0.001) (data not shown).

### Drinking habits of tea, coffee, etc.

As regards the frequency of drinking tea and suchlike, respondents were classified by their answers into those who drink scarcely, 1-3 cups /week, 4-6 cups/week, 1-2 cups/day, 3-4 cups/day and 5+ cups/day. Respondents in higher dose groups tended to drink Japanese green tea quite often, but not much black tea, coffee or oolong tea (data not shown). However, after adjustment for age-class at the time of response, this tendency became less marked with the exception of the habit of drinking black tea. If the answers for the black tea were grouped into two, i.e. those who drink at least 1-3 cups / week or more and those who drink scarcely, the frequency of the former group was significantly lower in higher dose groups after age class was adjusted for (p<0.001). For instances, the frequency of the habit of drinking black tea was 33.4% in the <10mSv dose group and 16.0% in the 100+mSv group, when the data of all age classes were combined.

### Specific occupational history other than the radiation work

**Figure 3.**
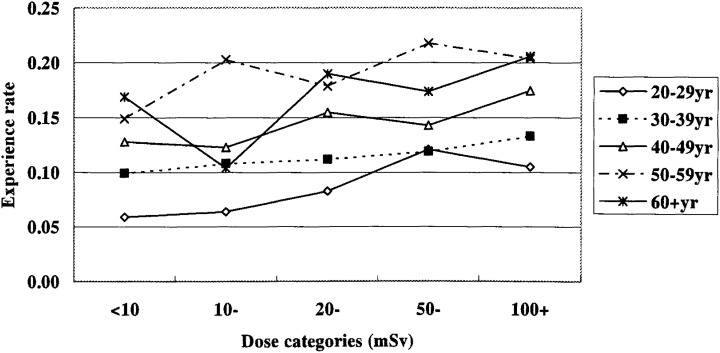
Percentages of male workers who have a history of special health checkups for harmful job by age class and dose category. Interaction of age and dose classes on the percentage; p=0.08. Trend with the doses; p<0.001 after adjusting for age classes.

Of the total respondents, 11.2% reported that they had taken part in a special compulsory health check program for workers engaged in specific hazardous jobs listed in the previous section. The percentage was greater in higher dose groups (Figure 3). The trend was statistically significant after adjustment for age classes with Mantel extension test (p<0.001).

Thirteen percent of respondents answered as having worked in some hazardous job. In detail, 6.1% had handled “organic solvents”, followed by 5.6% of exposure to “dust”, 1.8% to “asbestos” and so on, but it could not be determined whether those jobs were really of the kinds specified by law to be hazardous jobs. The tendency for respondents in higher dose groups to have a higher percentage of engagement in any one of these hazardous task was significant in all age classes (data not shown).

### History of medical radiation exposure

**Figure 4.**
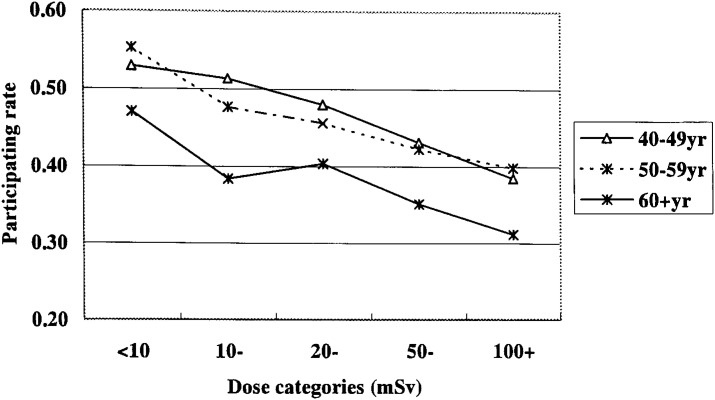
Percentages of male workers who underwent X-ray examination of the upper digestive tracts at the time of health checkup program offered by the company in the past year. Interaction of age and dose classes on the percentage; p=0.23. Trend with the doses; p<0.001 after adjusting for age classes.

Among those aged 40 years or more, 48.9% answered as having undergone X-ray examination of the upper digestive tract as part of the health checkup offered by their companies within the latest one year. The tendency of respondents in higher dose groups to have a lower rate of this examination was found to be extremely significant (Figure 4).

Other than this company health checkup program, some 36% replied that they had received “medical examinations at hospitals or other medical facilities”, and about 25% mentioned that they had also received “X-ray examination or other nuclear medicine procedures”. With respect to body sites, about 13% had received examinations of the “stomach and intestines”, and about 6% had CT examinations of the “chest and abdomen”. The rate of these various radiological examinations tended to be significantly lower in higher dose groups, again if limited to those aged 40 years or more (data not shown).

## DISCUSSION

The results of this survey showed that the habits of tobacco smoking and alcohol drinking were both more frequent among these workers as compared with the general population. According to the National Nutritional Survey conducted in 1999 by the Ministry of Health, Labor and Welfare,^15^ the percentage of current smokers among Japanese males was 56.3, 58.1, 57.7, 52.9 and 42.1%, and that of current drinkers was 34.0, 48.8, 60.6, 64.3 and 55.2%, in the age classes of 20-29, 30-39, 40-49, 50-59 and 60+ years, respectively. Compared with these figures, the percentage of smoking in the present study subjects was 5 to 12% higher and the drinking rate was 19 to 32% higher, though the definition of current drinker was slightly different between the National Survey and the present study. The higher smoking and drinking rates in the present population were, as will be discussed later, probably due to the difference found generally by occupational groups.

With regard to the other items of the questionnaire, comparative figures are scarcely available in the general population of Japan. For example, there have been no published data on the frequency of medical use of radiation. According to the recently conducted nation-wide survey of hospitals on the radiological examinations, male individuals underwent radiological examinations, including all kinds but that for the mass screening of cancers of various organs, 1.3 times and 1.6 times per year on the average in the age-classes of thirties and forties, respectively (K. Nishizawa, personal communication). However, contributions to these figures should come from those patients who contracted various diseases. They are not comparative to the corresponding figure, 0.25, in the present study.

In relationship to the cumulative doses of radiation, study subjects of higher dose groups significantly tended to be habitual tobacco smokers, have started smoking at a younger age, smoked a greater number of cigarettes, consumed a larger amount of alcohol drinks and less black tea, have been engaged more frequently in hazardous jobs specified by law, and have less frequently underwent radiological examinations such as X-ray mass examination of the upper digestive tracts.

We think that these results may be partly interpreted by their job backgrounds. Radiation doses received by those radiation workers are in most part generated at the time of periodic inspection of nuclear power plants. Workers of higher dose groups are generally skilled experts of a variety of jobs related with dismantling, inspecting, repairing and so forth of machines. Furthermore they are requested to be strictly trained of jobs in radiation controlled areas. Most of them are to be grouped to the skilled blue-collared workers. They are engaged in the radiation work for many years and tend to work for a long period by migrating among several nuclear facilities one after another. In contrast, those of lower dose groups are usually short term radiation workers who are hired for a short period and many of those with zero doses are white-collared workers who are actually not engaged in radiation works. This differential job background can be inferred from the average number of nuclear facilities in which the workers of different dose groups visit for radiation works in a year. For instances, the proportion of workers who were working in more than three sites per year was only 14.2% in the lowest dose class (<10mSv), but it was successively increased in higher dose classes reaching 69.4% in the highest dose class (100+ mSv)

According to the annual survey of tobacco consumption of Japanese general population in 1992,^16^ the smoking rate was different by occupational groups. In males, it was highest in sales and service workers (74.9%) and followed by blue-collared workers (69.0%). The lowest was in agricultural workers (54.8%) and managers and professional workers (55.7%). Other white collared workers were of an intermediate rate (57.8%). The present result of smoking rate in radiation workers is considered as a reflection of this general tendency. For alcohol drinking habit, though data were scarcely available for the general population, similar tendency would be speculated as for the variable drinking rate among different occupational groups. For the participation rate to the mass screening of gastrointestinal tract cancers, we also have no data as to its variation within the occupational groups in general. Still it seems reasonable to say that those people who are frequently moving from one place to another because of job should be hard to attend those health care programs constantly.

Epidemiological studies of radiation workers in other countries have scarcely been conducted with included information of various characteristics of study subjects which might play a role of confounders, except for their job related factors^17^ and socioeconomic status.^5^^,^^18^ So far, life-style of individual workers has never been studied simultaneously, as has been usually seen in a historic cohort study of occupational epidemiology.^19^ A case control study of lung cancer, which was conducted with including information of smoking habit, did not find any positive confounding effect of this factor in estimation of radiation risk.^20^ The present study has demonstrated for the first time that various life-style characteristics of radiation workers were highly correlated with their exposed radiation dose classes at least in Japan, although it was not conforming with the result of a cross-sectional study on the smoking habit of radiation workers in the United Kingdom.^21^

All the characteristics which showed a correlation with increasing doses in the present study, except for the black tea drinking habit, are known to be associated with cancer incidence and mortality based on the present state of epidemiological and biological knowledge of cancer.^8^^,^^9^^,^^11^^,^^12^^,^^22^ Therefore, it seems highly probable that the results of the radiation epidemiological study of those radiation workers (T. Iwasaki et al, under submission), which aimed risk assessment of low level radiation exposure, was confounded and influenced by these factors particularly for solid cancers. For example, that report demonstrated a strongly positive correlation of esophagus cancer mortality with radiation doses. For this cancer, on the other hand, strong synergistic effect of smoking and drinking habits has been consistently shown,^23^ so that the observed dose-response of mortality may partly, though not entirely, explained by the difference of smoking and drinking habits among dose classes, if assuming that the present study population was well representing the cohort population.

It should be remembered, however, that these individual factors in overall life-style habits alone do not necessarily function as the confounding factors. Instead, they may represent certain life-style characteristics of the study population. For example, dietary patterns are known to be quite different between smokers and non-smokers.^24^ The original purpose of the study for “percentage of those who underwent X-ray examinations” was to obtain information regarding the state of medical radiation exposure of this study population. However, the present results did not allow quantitative evaluation of medical radiation exposure. Instead, it is presumable that subjects in higher dose groups are typically people who either “hate to see doctors”, or are “too busy to visit clinics”. Although such life-style characteristics have not yet been proven to be really linked to cancer mortality, a thorough examination of relevant confounding life-style habits is imperative to improve future studies of radiation workers.
